# Salmon (*Salmo salar*) Side Streams as a Bioresource to Obtain Potential Antioxidant Peptides after Applying Pressurized Liquid Extraction (PLE)

**DOI:** 10.3390/md19060323

**Published:** 2021-06-03

**Authors:** Beatriz de la Fuente, Noelia Pallarés, Houda Berrada, Francisco J. Barba

**Affiliations:** Preventive Medicine and Public Health, Food Science, Toxicology and Forensic Medicine Department, Faculty of Pharmacy, Universitat de València, Avda. Vicent Andrés Estellés, 46100 València, Spain; beatriz.fuente@uv.es (B.d.l.F.); noelia.pallares@uv.es (N.P.)

**Keywords:** pressurized liquid extraction, salmon, side streams, peptides, protein, SDS-PAGE, antioxidant capacity, mycotoxins, heavy metals

## Abstract

The pressurized liquid extraction (PLE) technique was used to obtain protein extracts with antioxidant capacity from salmon muscle remains, heads, viscera, skin, and tailfins. A protein recovery percentage ≈28% was obtained for all samples except for viscera, which was ≈92%. These values represented an increase of 1.5–4.8-fold compared to stirring extraction (control). Different SDS-PAGE profiles in control and PLE extracts revealed that extraction conditions affected the protein molecular weight distribution of the obtained extracts. Both TEAC (Trolox equivalent antioxidant capacity) and ORAC (oxygen radical antioxidant capacity) assays showed an outstanding antioxidant activity for viscera PLE extract. Through liquid chromatography coupled with electrospray ionization triple time-of-flight (nanoESI qQTOF) mass spectrometry, 137 and 67 peptides were identified in control and PLE extracts from salmon viscera, respectively None of these peptides was found among the antioxidant peptides inputted in the BIOPEP-UMP database. However, bioinformatics analysis showed several antioxidant small peptides encrypted in amino acid sequences of viscera extracts, especially GPP (glycine-proline-proline) and GAA (glycine-alanine-alanine) for PLE extracts. Further research on the relationship between antioxidant activity and specific peptides from salmon viscera PLE extracts is required. In addition, the salmon side streams studied presented non-toxic levels of As, Hg, Cd, and Pb, as well as the absence of mycotoxins or related metabolites. Overall, these results confirm the feasible use of farmed salmon processing side streams as alternative sources of protein and bioactive compounds for human consumption.

## 1. Introduction

Salmon consumption has tripled since the 1980s, mainly because it is considered a healthy food due to its contents of polyunsaturated fatty acids, quality proteins, vitamins, and minerals [1,2]. The versatility of commercialized salmon products (i.e., fresh, frozen, smoked, fillet, canned, sushi, ready meals) is also related to a wide distribution, as well as an increased interest aroused by consumers and food industry [1,2]. At the same time, the salmon aquaculture sector has grown worldwide. In Europe, Atlantic salmon (*Salmo salar*) is currently the most important farmed species in volume and value, exceeding 1.3 million tons and 5 billion EUR in 2017 [3]. Since salmon has a great fillet yield, it is one of the most highly processed fishes [4]. As a result, 50% of complete fresh salmon has been estimated to correspond to side stream materials [5]. Therefore, a large amount of discards are available to develop high-added-value products, including those intended for human consumption. In this context, the nutritional characterization of several salmon processing side streams revealed that they are rich in protein (10–20%) and fat (20–30%) [5,6], which make them candidate substrates for protein and oil recovery. Salmon side streams also showed relevant levels of essential amino acids (21–35%) as well as oleic acid (39–42%) and omega-3 fatty acids (19–21%) [5,6]. In addition, peptides with functional and bioactive properties are also found in several marine side streams [7,8,9]. For instance, peptides from salmon trimmings and pectoral fins have exhibited antihypertensive and antioxidant activities [4,10]. Antioxidant peptides from the viscera of sardinella, black pomfret, and mackerel have also been reported [9]. Therefore, salmon side stream materials could be considered a promising source of valuable compounds from the European circular economy point of view [11].

The valorization of seafood discards has been gaining attention over the last years, as their nutritional and bioactive compounds can now be extracted more efficiently using green technologies [12,13]. Pressurized liquid extraction (PLE) is currently considered an environmentally friendly technique to recover bioactive compounds from food matrices, as water is the most preferred solvent for the extraction process [14]. PLE is based on the use of high pressure and temperature to improve the extraction performance [15,16]. The possibility of applying different extraction conditions has made PLE a useful tool to optimize the extraction of high-added-value compounds from a wide variety of matrices, including marine sources and related side streams. For instance, PLE-assisted extraction was recently used to obtain aqueous protein extracts with in vitro antioxidant capacity from several side streams of rainbow trout, sole, sea bass, and sea bream [17,18,19]. Protein extraction from macro- and micro-algae using PLE has been also investigated [20].

In addition to healthy nutritional properties, any starting material that can be used in the food industry must be free of potentially harmful substances. In this sense, farmed fishes can be exposed to mycotoxins from plant-based feed [21,22], as well as toxic metals from the aquaculture environment [23]. A wide range of ingredients is used in the formulation of Atlantic salmon feed [24]. Because an important protein fraction comes from soy, corn, canola, and pea meals, the occurrence of mycotoxins in fish tissues must be evaluated. In a similar way, heavy metals have been found in several side streams of different fish species [18,19,25]. Therefore, assessing the levels of toxic elements in all fish tissues is advisable.

The main objective of the present study was to apply, for the first time, PLE-assisted extraction as a sustainable technique to obtain antioxidant protein extracts from salmon processing side streams. Muscle remains, heads, viscera, skin, and tailfins of farmed salmon were selected in order to give added value to these underutilized raw materials. Protein recovery, SDS-PAGE profile, and antioxidant capacity were evaluated in extracts obtained from salmon discards. Peptide identification and bioinformatics analysis in terms of potential antioxidant activity were performed for salmon viscera extracts. In order to provide additional data on possible contaminants in farmed fish, the levels of As, Hg, Cd, and Pb, as well as the occurrence of mycotoxins, were also investigated. Overall, this study contributes to the current marine resources valorization approach, focusing on the possibilities of processing side streams from farmed salmon.

## 2. Results and Discussion

### 2.1. Total Antioxidant Capacity

**Figure 1:**
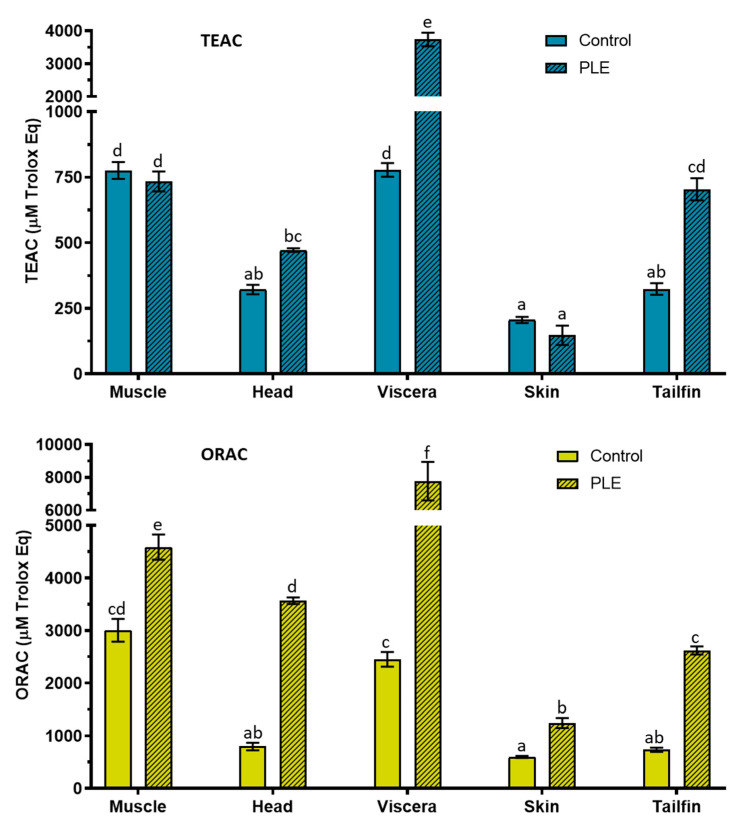
Total antioxidant capacity determined by TEAC and ORAC in control and PLE extracts from salmon muscle, head, viscera, skin, and tailfin. TEAC: trolox equivalent antioxidant capacity. ORAC: oxygen radical absorbance capacity. PLE: pressurized liquid extraction. µM Trolox Eq (micromolar trolox equivalent). Results of TEAC (n = 3) and ORAC (n =6) are expressed as mean ± standard deviation. Different lowercase letters in the bars indicate statistically significant differences (*p* < 0.05) among samples.

The results of total antioxidant capacity, determined using the Trolox equivalent antioxidant capacity (TEAC) and oxygen radical antioxidant capacity (ORAC) methods in control and PLE extracts of salmon side streams, are shown in Figure 1. TEAC values in PLE extracts were 734 ± 38, 472 ± 7, 3739 ± 209, 147 ± 37, and 704 ± 42 µM Trolox Equivalents (Eq) for muscle, head, viscera, skin, and tailfins, respectively, whereas TEAC values in the corresponding control extracts were 776 ± 32, 322 ± 18, 778 ± 26, 206 ± 12, and 324 ± 22 µM Trolox Eq. Regarding the ORAC assay, the values of total antioxidant capacity were higher in PLE extracts than in control extracts for all samples. ORAC values (µM Trolox Eq) in PLE extracts were 4586 ± 241 (muscle), 3567 ± 63 (heads), 7772 ± 1174 (viscera), 1244 ± 94 (skin), and 2620 ± 78 (tailfins), whereas control ORAC values were 3005 ± 217, 797 ± 73, 2451 ± 139, 599 ± 19, and 736 ± 39, respectively. Therefore, PLE-assisted extraction improved the antioxidant capacity (ORAC) compared to conventional extraction for all salmon side streams. The increases were 1.5-, 4.5-, 3.2-, 2-, and 3.6-fold for muscle, head, viscera, skin, and tailfins, respectively. As for TEAC, the antioxidant capacity of PLE extracts also increased compared to the controls for head (1.5), viscera (4.8), and tails (2.2), whereas the muscle and skin values remained without significant changes. The highest antiradical activity was observed in PLE extracts of viscera for both antioxidant assays. These results are slightly different to those obtained for PLE extracts of sea bass and sea bream by-products, in which muscle PLE extracts showed the highest values of antioxidant capacity determined by both TEAC and ORAC methods [18,19]. The antioxidant capacity of viscera PLE extracts from sea bass and sea bream were similar to those of head PLE extracts. These differences may be due to the fact that seabass and sea bream are a more closely related species compared to salmon.

On the other hand, the different antioxidant capacity exhibited by the protein extracts obtained is probably related to both the size and the amino acid composition of the protein fragments of each salmon side stream. Several authors have suggested that hydrophobic amino acids could contribute to the total antioxidant activity of protein fragments [7,9]. In this way, glycine and glutamic acid have been reported as the most abundant polar amino acids in salmon heads, skin, and viscera [5]. Hydrophobic amino acids such as alanine, proline, leucine, and valine were also found in relevant quantities. In addition, the molecular weight of fish peptides (0.5–1.5 kDa) has been associated with antioxidant properties [7,26]. According to this, the outstanding antioxidant capacity shown by PLE viscera extracts could mean the presence of bioactive peptides with some of the aforementioned amino acids in their sequence.

### 2.2. Protein Recovery Percentage

**Figure 2:**
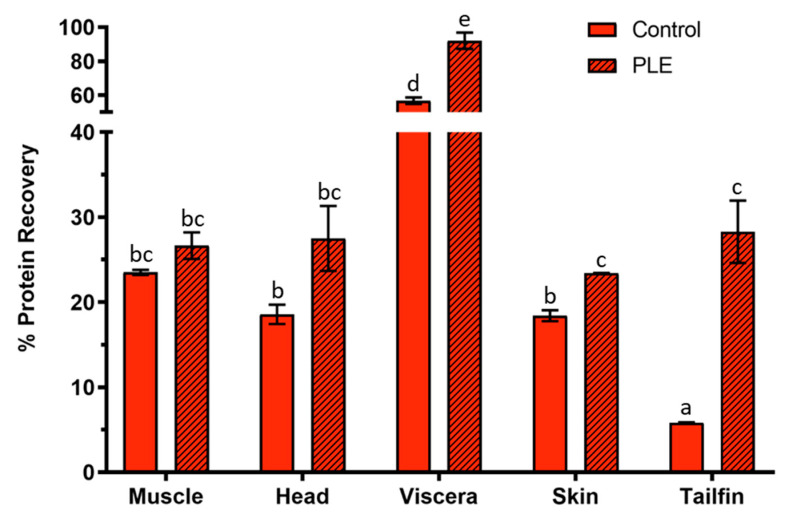
Percentage of protein recovery in control and PLE extracts from salmon muscle, heads, viscera, skin, and tailfin. PLE: pressurized liquid extraction. Results are expressed as mean ± standard deviation (n = 2). Different lowercase letters in bars indicate statistically significant differences (*p* < 0.05) among samples.

The results of protein recovery in control and PLE extracts from side streams of gilthead sea bream are shown in Figure 2. The percentage of protein recovery in PLE extracts of salmon muscle, head, viscera, skin, and tailfins were 26.65 ± 1.57, 27.50 ± 3.83, 92.03 ± 4.80, 29.39 ± 0.05, and 28.29 ± 3.66, respectively, whereas those of their corresponding control extracts were 23.51 ± 0.31, 18.57 ± 1.14, 56.76 ± 1.87, 18.41 ± 0.64, and 5.82 ± 0.63. Therefore, PLE improved the protein recovery for all side streams. The improvement in protein recovery was close to 1.5-fold for heads, viscera, and skin extracts. The tailfin extracts experienced a 5-fold increase with the PLE technique, whereas salmon muscle results were similar for both conventional stirring and PLE extraction. The best protein recovery was observed in viscera, consistent with previously observed protein recoveries in extracts of sea bass and sea bream side streams after applying PLE-assisted extraction [18,19]. Few food matrices or related side streams have been used for protein extraction by means of PLE. For instance, different seaweeds, as well as seeds from red pepper, showed protein recovery percentages about 5% and 50%, respectively [20,27].

### 2.3. Protein Molecular Weight Distribution

**Figure 3:**
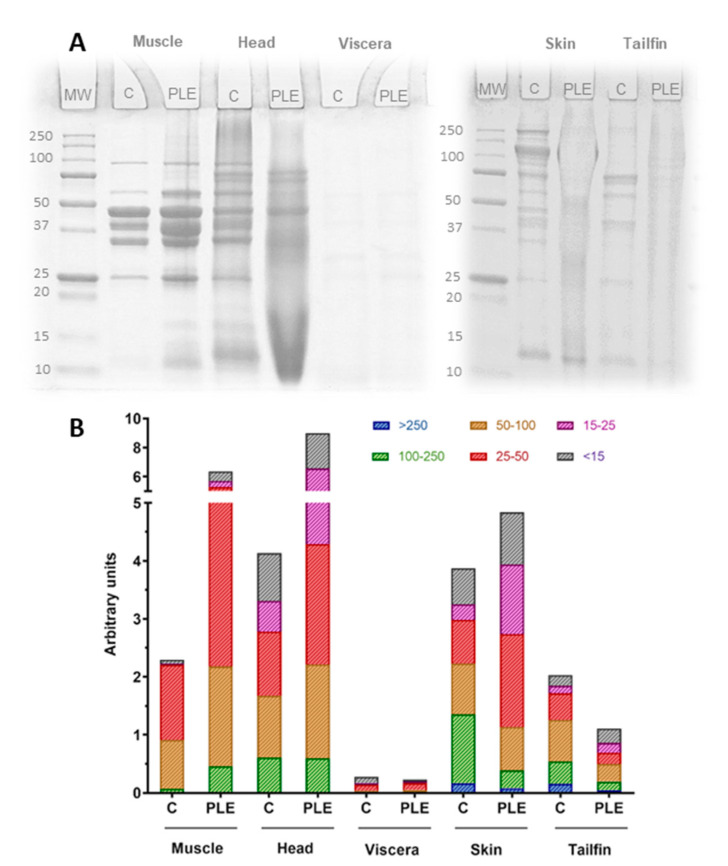
Protein molecular weight distribution of control and PLE extracts from salmon side streams. SDS-PAGE protein profiles (**A**) and molecular weight ranges for band areas (**B**). MW: molecular weight standard. C: control extract. PLE: extract obtained by means of pressurized liquid extraction.

The protein molecular weight distribution of salmon side stream extracts, obtained both through conventional stirring and PLE-assisted extraction, was provided by means of SDS-PAGE (Figure 3A). As can be seen in the images, the extracts presented different electrophoretic profiles. In general, these differences appeared to be related to both the type of side stream and the type of extraction process. In order to obtain the molecular weight of each band and also to group the areas of the bands by kDa ranges, the images of the gels were analyzed using ImageJ and GraphPad Prism Programs (Figure 3B). For muscle leftovers, clear bands from 9 to 108 kDa were observed in control and PLE extracts, which could be due to the fact that both extraction processes were carried out at room temperature. However, the differences in the width of the bands revealed that PLE extracts presented a greater amount of total protein fragments for all molecular weight groups. This behavior is in agreement with those previously reported for sea bass and sea bream muscle remains subjected to the same PLE and shaking extraction conditions [18,19]. Protein fragments of head control extracts showed several bands from 10 to 108 kDa, whereas the highest protein molecular weight for head PLE extracts was of 96 kDa. In addition, bands of 20–50 kDa in head control extracts were not found in head PLE extracts. In contrast, control and PLE extracts from salmon viscera exhibited the same protein molecular weight distribution (≤7–73 kDa) and few slight bands. The range of values was similar to that shown by sea bass and sea bream viscera extracts (8–61 kDa) [18,19].

Both skin and tailfin extracts presented wider molecular weight ranges (≈6–140 kDa) than muscle, heads, and viscera extracts. Furthermore, for both samples, several protein bands in control extracts did not appear in PLE extracts. According to the gel image analysis, bands in 25–50 and 75–125 kDa ranges from control skin extracts were not present in the corresponding PLE extracts. Similarly, the 10–30 kDa protein fragments in tailfin control extracts were not found in those of PLE. The protein molecular weight distribution of discards from Australian Atlantic salmon was evaluated previously [5]. The head and skin protein fragments were in the range of 25–250, whereas most of the viscera were below 10 kDa.

Based on these results, sodium dodecyl sulfate polyacrylamide gel electrophoresis (SDS-PAGE) revealed different protein profiles between the matrices studied. In addition, differences observed among control and PLE extracts for each side stream have shown that PLE-assisted extraction influenced the size of protein fragments obtained in the extracts. It should be noted that this electrophoretic technique provides additional information as to the total protein content. However, it does not allow the retention of peptides in the gel, which could be relevant to correlate the presence of peptides with the antioxidant capacity shown by the extracts.

### 2.4. Identification of Peptides in Viscera Extracts

As previously described, the salmon viscera extracts obtained through PLE-assisted extraction resulted the most interesting sample in terms of in vitro antioxidant capacity. Their TEAC and ORAC values not only stand out against the other salmon byproducts studied here, but also in comparison with previously investigated PLE protein extracts from sea bass and sea bream viscera. For this reason, PLE protein extracts from salmon viscera were selected for the identification of antioxidant peptides. Control viscera extracts were also screened in order to compare peptides extracted through PLE and under stirring conditions. Only peptides with a confidence percentage ≥ 90% have been reported.

A total of 137 peptides were identified in the PLE viscera extracts (Table 1). In contrast, 67 peptides were identified in the viscera control extracts (Table 2). Despite using the same viscera sample, only five peptides matched in both extracts (color marked in both tables). These data show that the extraction conditions used for PLE-assisted extraction influence the peptides obtained from salmon viscera.

A common method currently used to speculate about peptide function is through an amino acid homology alignment against a database of known functional peptide sequences. The antioxidant activity of the identified peptides was thus predicted using the BIOPEP-UWM database, which is a bioinformatics tool for searching among bioactive peptides, mainly derived from foods [28]. None of the peptides identified in salmon viscera extracts were found among the antioxidant peptides inputted in the BIOPEP-UMP database. Therefore, a new search based on the profiles of the potential biological activity of peptides was performed. BIOPEP-UWM analysis results exhibited several antioxidant small peptides encrypted in amino acid sequences of PLE (Table 3) and control (Table 4) viscera extracts, with some of them known to be derived from marine species. Throughout the entire structure of peptides, 19 different sequences of peptides with antioxidant activity were found in the PLE extract, whereas there were 12 in the control extract. Most of these potential antioxidant peptides were di- and tri-peptides. The sequence GPP was found in 15 peptides of the PLE extract, followed by GAA, which was found in five peptides. These sequences could be responsible for antioxidant activity, since antioxidant peptides from marine resources have been described to contain hydrophobic acids such as glycine (G), proline (P), and alanine (A) [8,9,29]. Furthermore, salmon antioxidant peptides from the pectoral fin (FLNEFLHV) and trimmings (GGPAGPAV, GPVA, PP, GP) have been reported [10,30]. Several antioxidant peptide sequences from the viscera of sardinella (LHT, LARL, GGE), black pomfret (AMT6GLEA), and mackerel (ACFL) have also been identified [9].

**Figure 4:**
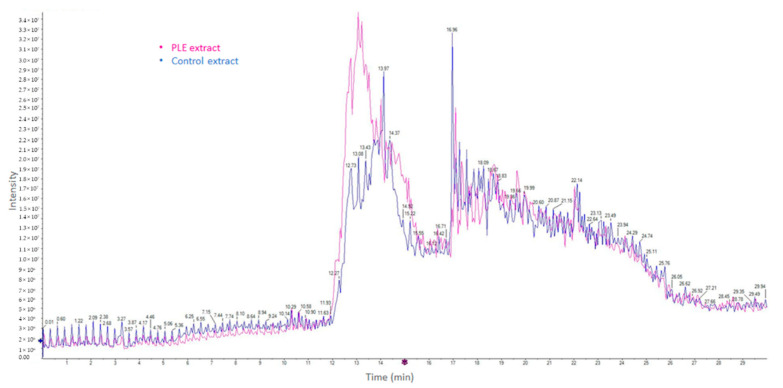
Chromatogram of total ion counts of salmon viscera protein extracts, obtained through conventional stirring and pressurized liquid extraction (PLE).

In addition to specific amino acids, peptides derived from fish sources, especially in the range of 0.5–1.5 kDa, have been assumed to be a key factor in terms of antioxidant activity [26]. The molecular weight of peptides in control viscera extracts ranged from 0.63 to 2.44 kDa (Table 4), whereas for viscera PLE extracts, the molecular weight of peptides was 0.67–2.60 kDa (Table 2). However, there was a greater amount of small peptides in the PLE extract. As can be seen in Figure 4, a higher intensity of analytes with shorter retention times was observed for the viscera PLE extract, which in the case of peptides usually corresponds to more polar and/or smaller compounds.

According to these results, both the specific amino acid sequences encrypted in the identified peptides and a molecular weight below 1.5 kDa could be related to the antioxidant capacity exhibited by the PLE extract obtained from salmon viscera.

### 2.5. Determination of Heavy Metals and Mycotoxins in Salmon Side Streams

The concentrations of As, Hg, Cd, and Pb in salmon muscle, heads, viscera, skin, and tailfins are shown in Table 5. Mean concentration ranges, expressed as µg/g of wet weight (ww), were 0.4186–0.6922, 0.0095–0.0408, 0.0004–0.0104, and 0.0071–0.0859 for As, Hg, Cd, and Pb, respectively. For all salmon side streams, the most abundant element was As, whereas the lowest concentration was observed for Cd. There is a lack of information in the literature on heavy metal contents in salmon discards. For instance, one study reported liver Hg accumulations in four wild species of Pacific salmon [31]. The results (0.120–0.192 µg/g, ww) were higher than those found in the present study for viscera samples, which include more organs than the liver. The contents of As, Hg, Cd, and Pb in several fish side streams of sea bass, sea bream, and meager have also been described [18,19,23,25]. The arsenic levels in the viscera (1.867–2.587 µg/g, ww) of these fish species were higher than those in the salmon viscera.

The data available on toxic elements in fish usually refer to edible muscle due to the potential health risk for consumers. In this sense, levels of Cd and Pb in 21 samples of smoked salmon from a Polish market were determined [32]. The results were on the order of 0.0040–0.0196 µg/g (ww) for Cd and 0.0109–0.1559 µg/g (ww) for Pb, both of which are considered safe for consumers. In addition, As, Hg, Cd, and Pb contents in fresh salmon muscle were evaluated [33,34]. It should be noted that the limits for heavy metals in fish side streams are not currently regulated. Therefore, the safety assessment could be based on the limit values established for edible muscles of fish (µg/g): 13.5 for As, 0.5 for Hg, 0.05 for Cd, and 0.30 for Pb [23,25,35]. According to this, the toxic elements analyzed in all salmon side streams in this study are below the limits set by authorities and could be considered safe for consumers in terms of As, Hg, Cd, and Pb content.

Nostbakken et al. [33] showed a trend towards a decrease in As and Hg content in farmed Atlantic salmon, which was related to the decline in the use of fish meal and fish oil in commercial fish feed. However, the replacement of marine ingredients by others of plant origin can lead to the presence of contaminants such as mycotoxins in both aquafeeds and fish tissues. In this way, Bernhoft et al. [36] conducted a toxicokinetic study of deoxynivalenol (DON) and ochratoxin A (OTA) mycotoxins in farmed salmon fed with contaminated feeds for 8 weeks. The authors observed an even distribution in the liver, kidney, brain, skin, and muscle for DON, as well as a distribution mainly in the liver and kidney for OTA. According to this, the possible occurrence of mycotoxins in the muscle, head, viscera, skin, and tailfin of farmed salmon was investigated in the present study. Through a simultaneous multi-mycotoxin evaluation using a non-targeted screening approach, no mycotoxins or related metabolites were identified in salmon side streams. These results are in agreement with those found by Nácher-Mestre et al. [37,38] on the carry-over of common and emerging mycotoxins from feeds to edible parts of farmed Atlantic salmon fed with high plant-based diets. In addition, there was no presence detected of several mycotoxins, such as aflatoxins, fumonisins, enniatins, or ochratoxin A, in smoked salmon and raw salmon sushi commercial products [39].

## 3. Materials and Methods

### 3.1. Reagents

AAPH (2,2′-azobis (2-amidinopropane)) (Acros Organics), sodium phosphate dibasic, sodium chloride, potassium dihydrogen phosphate, potassium sulphate, TRIS (ultrapure), glycine (proteomics grade), ortho-boric acid, and methanol (HPLC grade) were obtained from VWR International Eurolab S.L. (Barcelona, Spain). Trizma^®^ base, ABTS (2,2′-azinobis (3-ethylbenzothiazoline 6-sulfonic acid)), DTT (DL-Dithiothreitol), Trolox^®^ (6-hydroxy-2,5,7,8-tetramethylchroman-2-carboxylic acid), fluorescein sodium salt, formic acid (reagent grade ≥ 95%), and diatomaceous earth (Hyflo^®^ Super Cel^®^) were provided by Sigma-Aldrich (Steinheim, Germany). Sodium hydroxide, glacial acetic acid, and sulfuric acid were supplied by Fisher Scientific (Madrid, Spain). SDS (sodium dodecyl sulfate) and nitric acid (65% p/p) were purchased from Panreac (Barcelona, Spain). Bromophenol blue indicator (ACS reagent), acetonitrile (HPLC grade), trifluoroacetic acid, acetone, and glycerol were provided by Merck (Darmstadt, Germany). Absolute ethanol was obtained from J.T. Baker (Deventer, The Netherlands), Octadecyl C18 sorbent was obtained from Phenomenex (Madrid, Spain), and anhydrous magnesium sulfate (99.5% min powder) was obtained from Alfa Aesar (Karlsruhe, Germany). Deionized water with a resistivity of >18 MΩ/cm was obtained through a Milli-Q SP^®^ Reagent Water System (Millipore Corporation Bedford, MA, USA).

### 3.2. Raw Material and Sample Preparation

Whole salmon fish (*Salmo salar*) from Norwegian aquaculture were purchased in a local market in Valencia (Spain) during different weeks of June 2019. They were immediately transported to the laboratories of the University of Valencia under refrigerated conditions. Individual salmon were dissected as a simulation of fish processing for human consumption. Then, muscle leftovers, complete heads, viscera, flesh-free skin, and tailfins were placed separately inside aluminum containers and frozen at −80 °C for 48 h. Next, they were freeze-dried (LABCONCO, 2.5. FREE ZONE, USA) for 72 h, and keep in a desiccator until reaching a constant weight. Then, water content was determined gravimetrically. The moisture percentages were 67.61% ± 1.04%, 61.66% ± 2.52%, 52.31% ± 1.98%, 45.04% ± 1.60%, and 45.63% ± 0.71% for muscle remains, heads, viscera, skin, and tailfins, respectively. Similar values for salmon head, viscera, and skin were reported by Aspevik et al. [6] and He et al. [5]. Each type of sample was ground in an analytical mill (A11 basic IKA^®^ WERKE, Staufen, Germany) and stored at −25 °C until the extraction process and the determination of possible food contaminants.

### 3.3. Pressurized Liquid Extraction (PLE) Process

Antioxidant protein extracts from salmon side stream materials were obtained using an accelerated solvent extractor ASE 200 Dionex (Sunnyvale, CA, USA) equipped with a solvent controller. Dried samples were mixed with diatomaceous earth before being introduced into 22-mL stainless steel cells with a glass fiber filter placed in the end part. The standard operation parameters were as follows: preheating period (1 min), heating period (5 min), and flush volume (60%), and nitrogen purge (145 psi for 1 min). The extractions were performed under a pressure of 1500 psi with distilled water as a solvent. The pH, temperature, and time conditions for PLE-assisted extraction were selected based on the optimization of the extraction conditions to obtain antioxidant protein extracts from sea bass side streams [18]: pH 7, 20 °C, 5 min for muscle; pH 4, 60 °C, 15 min for heads; pH 7, 50 °C, 15 min for viscera; pH 7, 55 °C, 5 min for skin; and pH 7, 60 °C, 15 min for tailfins. For all samples, control extracts were also carried out in parallel by stirring for 30 min with distilled water at room temperature. Both types of extractions were performed at least in duplicate. The extracts obtained were homogenized individually, divided into several replicates and stored at −25 °C for subsequent analyses. Protein recovery, protein molecular weight distribution, and total antioxidant capacity were evaluated and compared (PLE vs control extracts).

### 3.4. Evaluation of Total Antioxidant Capacity

#### 3.4.1. Trolox Equivalent Antioxidant Capacity Assay (TEAC)

The TEAC assay measures the inhibition of the radical cation ABTS^+^ by antioxidant compounds, which is compared to the activity of a reference antioxidant standard (Trolox). The spectrophotometric method proposed by de la Fuente et al. [18] was used. ABTS reagent (7 mM) and K_2_S_2_O_8_ (140 mM) were mixed and maintained at room temperature in darkness for 16 h to generate the ABTS^+^ stock solution. Then, it was diluted in ethanol until an absorbance of 0.700 ± 0.020 at 734 nm and 30 °C to obtain the ABTS^+^ working solution. Proper dilution of each fish extract to achieve a percentage of absorbance inhibition of approximately 50% was required. A range of Trolox standard solutions (0–300 μM) were prepared. The absorbance of 2 mL of ABTS^+^ working solution was considered the initial point of reaction (A_0_). Then, 100 μL of diluted extracts or Trolox standards were added immediately. After 3 min of reaction, the absorbance was measured and considered the final point (A_f_). All measures were conducted in a thermostatized UV–vis spectrophotometer. The percentages of absorbance inhibition were calculated using the following equation: 1 − (A_f_/A_0_) × 100 and were compared to the Trolox standard curve. The results were expressed as μM Trolox Equivalents.

#### 3.4.2. Oxygen Radical Absorbance Capacity Assay (ORAC)

The ORAC assay measures the scavenging of the peroxyl radical AAPH by antioxidant compounds. The fluorometric method described by de la Fuente et al. [18] was applied. Sodium fluorescein (0.015 mg/mL), AAPH radical solution (120 mg/mL), and Trolox standard solution (100 μM) were prepared with phosphate buffer (75 mM, pH 7). Adequate diluted extracts were required. The operating conditions for the final reaction consisted of 50 μL of diluted extract, Trolox standard or phosphate buffer (blank), 50 μL of fluorescein, and 25 μL of AAPH incubated at 37 °C in a Multilabel Plate Counter VICTOR3 1420 (PerkinElmer, Turku, Finland). Fluorescence filters for an excitation wavelength (485 nm) and an emission wavelength (535 nm) were selected. The fluorescence was recorded every 5 min over 60 min, where the fluorescence in the assay was less than 5% of the initial value. Differences of areas under the fluorescence decay curve (AUC) between the blank and the sample over time were compared and the results were expressed as μM Trolox Equivalents.

### 3.5. Determination of Protein Recovery

The total nitrogen content in salmon side stream materials and extracts obtained by conventional stirring and PLE-assisted extraction was determined using the Kjeldahl method [40]. The total protein content was calculated based on the total nitrogen values and the protein–nitrogen conversion factor (6.25) for fish and fish side streams. Then, the following formula was applied for protein recovery: (protein in extract/protein in side stream) × 100.

### 3.6. Molecular Weight Distribution of Protein Fragments

SDS-PAGE was used to investigate the protein molecular weight distribution of both control (stirring) and optimal (PLE) extracts from salmon side stream materials. Acetone was added to the extracts at a 4:1 ratio (*v/v*) and they were mixed by means of a vortex. For protein precipitation, the mixture was centrifuged at 11,000 rpm, 4 °C, and 10 min. The supernatant was then removed and the pellet was dissolved and distilled. Afterwards, equal volumes of SDS-PAGE sample buffer solution (62.5 mM Tris-HCl (pH 6.8), 2% SDS, 20% glycerol, 0.01% bromophenol blue, and 50 mM dithiothreitol) and protein solution were mixed and heated in a thermoblock (95 °C, 5 min). Next, 10 μL were loaded onto 8–16% Mini-PROTEAN^®^ TGX™ Precast gels (Bio-Rad). The electrophoresis was performed using a Mini-PROTEAN^®^ Tetra Cell (Bio-Rad) under a constant voltage of 80 V for 120 min. The running buffer consisted of Trizma^®^ base (25 mM), glycine (192 mM), and SDS (0.1%). The gels obtained were stained in Coomassie brilliant blue R-250 (0.125%) and destained through a solution of water:methanol:acetic acid (70:20:10) until the background was as clear as possible. In order to estimate the molecular weight of protein bands obtained in the electrophoretic gels, a standard molecular weight of protein bands (5–250 kDa, Precision Plus Protein™, Bio-Rad) was used. The images of the gels were also evaluated using ImageJ^®^ software, a public domain digital image processing program developed at the National Institutes of Health (NIH). For a better visualization of protein bands, background subtraction and 8-bit format were selected.

### 3.7. Identification of Peptides in Viscera Extracts

#### 3.7.1. Sample Preparation

The salmon viscera extracts obtained through shaking and PLE were frozen and lyophilized. Freeze-dried samples (100 mg) were resuspended in MilliQ water (200 µL). Then, 200 µL of acetonitrile (ACN) were added and the mixture was kept overnight at 4 °C for protein precipitation. Next, samples were centrifuged at 5000 rpm for 5 min and the supernatants, which contained soluble peptides, were dried in a speed vacuum (Eppendorf, Hamburg, Germany). The resulting pellets were dissolved in 27 µL of aqueous solution, containing 2% ACN and 0.1% trifluoroacetic acid (TFA), and sonicated for 5 min. Afterwards, 0.5 µL of sample solution was diluted with 6 µL water with ACN (0.2%) and TFA (0.1%).

#### 3.7.2. Mass Spectrometry Analysis

Peptides were analyzed in a nanoESI qTOF mass spectrometer (6600plus TripleTOF, ABSCIEX, Framingham, MA, USA). A total of 5 µL of sample was loaded onto a trap column (ChromXP C18, 3 μm 120 Å, 350 μm, 0.5 mm; Eksigent) and desalted with 0.1% TFA at a flow rate of 5 µL/min for 5 min. The peptides were then loaded onto an analytical column (3µ C18-CL 120, 0.075 × 150 mm; Eksigent) equilibrated in 5% ACN and 0.1% TFA. Elution was carried out with a linear gradient from 7% to 40% B in A for 45 min. (A: 0.1% formic acid (FA); B: ACN, 0.1% FA) at a flow rate of 300 nL/min.

Sample was ionized by applying 3.0 kV to the spray emitter at 175 °C. Analysis was performed in a data-dependent mode. Survey MS1 scans were acquired from 350–1400 *m/z* for 250 ms. The quadrupole resolution was set to ‘LOW’ for MS2 experiments, which were acquired 100–1500 *m/z* for 25 ms in ‘high sensitivity’ mode. The following switch criteria were used: charge: 2+ to 4+; minimum intensity; 250 counts per second (cps). Up to 100 ions were selected for fragmentation after each survey scan. Dynamic exclusion was set to 15 s. The system sensitivity was controlled by analyzing 0.5 µg of K562 trypsin digestion (Sciex). In these conditions, 2230 proteins were identified (FDR <1%) in a 45 min gradient.

#### 3.7.3. Data Analysis

After LC-MS/MS, the identification of peptides was carried out with the software ProteinPilot v5.0 search engine (AB SCIEX). ProteinPilot default parameters were used to generate the peak list directly from 6600 plus TripleTOF wiff files. The Paragon algorithm [41] in ProteinPilot v 5.0 was used to search against the Swiss Prot (Inr 200602) and Uniprot Chordata (Inr 2007721) protein sequence databases with the following parameters: none digestion, none cys-alkylation, taxonomy non restricted, and the search effort set to thorough.

The BIOPEP-UWM database was used in the search for similar previously identified sequences showing antioxidant activity (http://www.uwm.edu.pl/biochemia/index.php/pl/biopep accessed on 28 April 2021). The search option “profiles of potential biological activity” was then employed, in which antioxidant activity was selected.

### 3.8. Analysis of Heavy Metals in Salmon Side Stream Materials

The presence of As, Hg, Cd, and Pb in side stream materials of farmed salmon was studied. Muscle, heads, viscera, skin, and tailfins were mineralized in a microwave oven (MARS, CEM, Vertex, Spain). Approximately 0.30 g of sample was placed in a Teflon reactor vessel. Next, 1 mL of H_2_O_2_ (30% *v/v*) and 4 mL of HNO_3_ (14M) were added and the digestion was conducted under a microwave irradiation power of 800 W at 180 °C for 15 min. The digested samples were left to cool at room temperature. After eliminating the nitrogenous vapor, they were filtered and brought up to volume with distilled water.

The identification and quantification of toxic metals was carried out using an inductively coupled plasma spectrometer mass detector (ICP-MS, Agilent model 7900). The analytical conditions were as follows: carrier gas (1.07 L/min), Ar gas flow (15.0 L/min), reaction gas (He), RF power (1550 W), nebulizer pump speed (0.10 rps), and RF matching (1.80 V). To correct matrix-induced signal fluctuations and instrumental drift, internal standard solutions of ^72^Ge, ^103^Rh, and ^193^Ir (ISC Science) at 20 µg/g were used. For the quantification of As, Cd, and Pb, standard calibration curves from 0 to 1000 µg/L were used. As for the quantification of Hg, a standard calibration curve from 0 to 100 µg/L was utilized. Limits of detection (LODs) were calculated according to the following equation: LOD = 3sB/a where “3sB” is 3 times the standard deviation at zero concentration and “a” is the slope of the calibration curve. LOD values obtained for As, Hg, Cd, and Pb were 0.012, 0.0015, 0.004, and 0.0015 µg/L, respectively. The concentrations of heavy metals in the digested blank (distilled water) were subtracted from the values of samples. The results were expressed as µg of element/g of side stream material in wet weight. To confirm the accuracy of the method, the fish protein powder DORM-3 was used as the Certified Reference Material for Trace Metals. It was prepared and analyzed simultaneously to the salmon samples. The recovery percentages were 98%, 86%, 76%, and 77% for As, Hg, Cd, and Pb, respectively.

### 3.9. Analysis of Mycotoxins in Salmon Side Stream Materials

High-performance liquid chromatography coupled with electrospray ionization-quadrupole-time of flight-mass spectrometry (LC-ESI-qTOF-MS) was employed to investigate the occurrence of mycotoxins in salmon side stream materials. An Agilent 1200-LC system (Agilent Technologies, Palo Alto, CA, USA) equipped with a Gemini^®^ column NX-C18 (3 µM, 150 × 2 mm ID) (Phenomenex), as well as a vacuum degasser, binary pump, and autosampler, were used to achieve the chromatographic separations The mobile phases consisted of acidified (0.1% of formic acid) water (A) and acetonitrile (B). A gradient program of 50% B (0–6 min); 100% B (7–12 min); and 50% B (13–20 min) was applied. Samples (5 µL) were injected at a flow rate of 0.2 mL/min. Mass spectrometry (MS) analysis was carried out using a 6540 Agilent Ultra-High-Definition-Accurate-Mass-q-TOF-MS coupled to the HPLC, equipped with an Agilent Dual Jet Stream electrospray ionization (Dual AJS ESI) interface in positive and negative ionization modes. The operational conditions were as follows: nebulizer pressure (50 psi); capillary voltage (3500 V); fragmenter voltage (160 V); scan range (*m/z* 50–1500); drying gas temperature (370 °C); and nitrogen drying gas flow (12.0 L/min). Automatic MS/MS experiments were performed under the following collision energy values: *m/z* 100, 30 eV; *m/z* 500, 35 eV; *m/z* 1000, 40 eV; and *m/z* 1500, 45 eV. For data acquisition and integration, Mass Hunter Workstation software was used.

The QuEChERS procedure to extract mycotoxins from fish discards, previously reported by de la Fuente et al. [18], was applied. Approximately 3 g of salmon samples were mixed with 30 mL of acidified water (2% formic acid) in an orbital shaker (IKA KS 260) for 30 min. Then, 10 mL of acetonitrile were added and the mixture was stirring again for 30 min. Next, 8 g of MgSO_4_ and 2 g of NaCl were added to the mixture, vortexed for 30 s and centrifuged at 4000 rpm for 10 min. Afterward, 0.1 g of Octadecyl C18 sorbent and 0.3 g of MgSO_4_ were mixed with 2 mL of supernatant. Additional shaking and centrifugation under the same conditions as reported previously were performed. The supernatant was then filtered (13 mm/0.22 μm nylon filter) and 20 μL were injected into the LC-ESI-qTOF-MS system.

### 3.10. Statistical Analysis

Experimental data were subjected to one-way analysis of variance (ANOVA) to determine the significant differences among samples. Tukey’s honestly significant difference (HSD) multiple range test, at a significance level of *p* < 0.05 was applied. Statistical analyses were performed with Statgraphics Centurion XVI.I software (Statpoint Technologies, Inc., The Plains, VA, USA).

## 4. Conclusions

The Pressurized Liquid Extraction (PLE) technique allowed us to obtain, for the first time, protein extracts with in vitro antioxidant capacity from Atlantic salmon processing side streams. PLE-assisted extraction influenced the size of the protein fragments obtained in the extracts, since extracts from muscle leftovers, heads, viscera, skin, and tailfins showed different SDS-PAGE profiles.

Both the highest protein recovery percentage (92%) and the highest antioxidant capacity were observed in the viscera PLE extract. As 40% of the peptides identified in the PLE extract contained small peptide sequences with known antioxidant activity, salmon viscera could be considered an interesting source of antioxidant peptides. Further research on the relationship between antioxidant activity and specific peptides from salmon viscera PLE extract is required.

The levels of toxic metals (As, Hg, Cd, and Pb) and the absence of mycotoxins in salmon processing side streams contribute not only to increasing the limited data in the literature about these contaminants in farmed fish, but also provide information about their safety as candidates for use in the food industry.

## Figures and Tables

**Table 1:** Peptides identified in salmon viscera extract obtained through pressurized liquid extraction.

Protein of Origin of the Identified Peptide	Sequence	Obs MW	Obs *m/z*	Theor z
Collagen alpha-2(I) chain	GESGPTGNGGPVGA	1155.52	578.77	2
Collagen alpha-2(I) chain	GPAGPHGPPG	842.4	422.21	2
Collagen alpha-2(I) chain	SGETGSAGITGPAGPR	1413.68	707.85	2
Uncharacterized PE-PGRS family protein	GGNGGAGGAGGNGGAGGLGG	1370.62	686.32	2
Collagen alpha-3(V) chain	GIPGPLGPL	819.45	410.73	2
Collagen alpha-3(V) chain	GIPGPLGPLGP	973.52	487.77	2
Collagen alpha-3(V) chain	GPAGHPGPPG	842.4	422.21	2
Collagen alpha-1(I) chain	GETGPAGPAG	812.4	407.21	2
Collagen alpha-1(I) chain	GLPGSPGPAGEAGK	1193.6	597.81	2
Glycine-rich protein DOT1	GGGGGHGGGAGGGGGGGPGG	1292.58	647.3	2
Collagen alpha-4(IV) chain	GPIGPLGPLGP	973.52	487.77	2
Probable heat shock protein ssa1	PGGAPGGMPGGAP	1021.47	511.74	2
WAG22 antigen	PAGTAAGGAGGAGGAPGL	1308.6	655.31	2
Collagen alpha-1(I) chain	GETGPAGPAG	812.4	407.21	2
Histone H2A	AQGGVLPNIQ	995.54	498.78	2
60 kDa heat shock protein, mitochondrial	VGGTSDVEVNEK	1232.58	617.3	2
Collagen alpha-1(XXII) chain	GYAKDGLPGIPGPQGET	1655.76	828.89	2
Filamin-A	VITPEEIVDPNVDEH	1704.81	569.28	3
Glycine-rich cell wall structural protein	GGGEGYGGGGANGGGY	1285.6	643.81	2
Fumarylacetoacetase	IGVAIGDQILDLSVIK	1652.97	827.49	2
Pulmonary surfactant-associated protein A	GPLGPPGGMPGH	1072.53	537.27	2
Collagen alpha-1(I) chain	GETGPAGPAG	812.4	407.21	2
Collagen alpha-4(IV) chain	GPPGLPGPPGPPGHKGF	1607.77	804.89	2
WAS/WASL-interacting protein family member	GGGGGGGGGGGGSGGNFGGGGPP	1586.64	794.33	2
Adenylate cyclase type 10	GRVNIQDLQKNKFLMRANT	2245.16	749.4	3
Forkhead box protein K1	QPPPGPPPPPP	1076.55	539.28	2
Exocyst complex component SEC5	ALMILIVVHSECFR	1629.91	815.96	2
tRNA dimethylallyltransferase	EAARDGWPAL	1084.53	543.27	2
Orotidine 5’-phosphate decarboxylase	RPAGAEAGDQK	1098.54	550.28	2
Homogentisate 1,2-dioxygenase	GPIGSNGLANPR	1152.52	577.27	2
Wolframin	NTAPLGPSCPQPPPAP	1508.68	755.35	2
Keratin, type II cytoskeletal I	TALGGAAGGMGGGGGMGGGM	1538.63	770.32	2
Integrin-linked-kinase-associated- serine/				
threonine phosphatase 2C	GLPPAGSGNSGSLATSGS	1515.64	758.83	2
Collagen alpha-4(IV) chain	ACAGMIGPPGPQGFP	1399.63	467.55	2
Collagen alpha-3(V) chain	GIPGPLGPLGP	973.52	487.77	2
Fatty acid-binding protein, liver	AIGLPDDLIQK	1181.67	591.84	2
Actin-related protein 3	VIDSGDGVTH	998.47	500.24	2
Collagen alpha-1(I) chain	GAPGPVGPAGKGETGPAGPAGPAG	1925.86	963.94	2
Chaperone protein DnaK	QAGEGGAGAGAGAAG	1100.52	551.27	2
Collagen alpha-2(V) chain	GNPGPLGPIGP	974.52	488.27	2
Collagen alpha-1(XVIII) chain	LPGPPGPPGPPGPRGYPG	1665.79	833.9	2
POTE ankyrin domain family member E	VMDSGDGVTH	1016.43	509.22	2
Uncharacterized protein SE_1560	GPLVLVDTDDL	1155.61	578.81	2
Serum albumin 1	AIQPDTEFTPPELDASS	1816.84	909.43	2
Phosphoenolpyruvate guanylyltransferase	SLAMLNDVLVAL	1257.73	629.87	2
Collagen alpha-1(I) chain	AGPPGADGQPGAK	1164.55	583.28	2
DNA (cytosine-5)-methyltransferase 3A	DPASPNVATTP	1068.5	535.26	2
Protein Shroom4	SQAPESHESRTGL	1397.61	699.81	2
Ataxin-2 homolog	PAGGGPQPAFTPP	1192.56	597.29	2
Magnesium-chelatase 38 kDa subunit	QSGENVVERDGL	1301.6	651.81	2
Uroporphyrinogen decarboxylase	DVAVQGNLDPL	1139.61	570.81	2
Collagen alpha-1(I) chain	AGAQGAPGPAGPA	1021.47	511.74	2
Collagen alpha-3(V) chain	GIPGPLGPLGP	973.53	487.77	2
Actin-related protein 3	DSGDGVTH	786.32	394.17	2
tRNA-N6-adenosine-threonylcarbamoy				
ltransferase	LSLVVSGGHTELVL	1422.69	712.35	2
Calpain-12	AGTGAGGPQ	714.2	358.11	2
Collagen alpha-1(XVII) chain	QNLVGPPGPPGPPGVSGD	1623.77	812.89	2
Fumarylacetoacetase	IGVAIGDQILDLSVIK	1652.97	552	3
Probable aquaporin PIP2-6	DINAGGGACASVGLL	1316.67	659.34	2
60 kDa chaperonin	AAVEEGIVAGGGTAF	1347.58	674.8	2
Arginine kinase	KGDRFLEAAGVNKLWPE	1928.92	965.47	2
Collagen alpha-2(I) chain	GETGSAGITGPAGPR	1326.65	664.33	2
Cytoplasmic dynein 1 light intermediate chain 1	TGSPGGPGVSGGSPAGGAG	1425.64	713.83	2
Collagen alpha-2(I) chain	RGDGGPPGVTGFPGAA	1411.63	706.82	2
Collagen alpha-1(I) chain	AKGDTGAPGAPGSQGAP	1437.68	719.85	2
Zinc finger protein 831	ESEGEGGPGPGPGVAGAEP	1649.78	550.93	3
Collagen alpha-2(IV) chain	PGEKGDAGLPGLSGK	1363.64	682.83	2
Collagen alpha-2(I) chain	GPTGNGGPVGA	882.42	442.22	2
Translation initiation factor IF-2	GGGGGAPGRPGGGGGGGGAP	1405.65	703.83	2
Collagen alpha-2(I) chain	GPAGPHGPP	785.38	393.7	2
Serine/threonine-protein kinase ATG1	ESNMFVSEYL	1217.56	609.79	2
ATP-dependent RNA helicase DBP7	REGKWDIHATT	1312.67	657.34	2
Nucleoside diphosphate kinase B	ETNPADSKPGSI	1214.58	608.3	2
Glucosyl-3-phosphoglycerate synthase	VAGDLAGGRAPGALP	1320.64	661.33	2
Collagen alpha-6(IV) chain	VGPLGPSG	682.33	342.17	2
Collagen alpha-3(V) chain	GIPGPLGPLGP	973.53	487.77	2
5’-3’ exoribonuclease 2	NNGGGGGGYGGQP	1090.51	546.26	2
PE-PGRS family protein PE_PGRS30	NGGAAGLIGNGGAGGAGGAGGAG	1639.72	820.87	2
Protein FAM81B	DTNVNKSASPTATAEEQPVEP	2184.09	1093.05	2
E3 ubiquitin-protein ligase TOPORS	DQGLFMGPSTSGAAANR	1679.7	560.91	3
(R)-2-hydroxyglutaryl-CoA-dehydratase				
activating ATPase	GIADKQMSELSCHA	1488.7	745.36	2
Uncharacterized TPR repeat-containing				
protein At1g05150	DALGLELNADE	1158.57	580.29	2
Collagen alpha-1(I) chain	DGNPGLPGPPGPPGPPG	1492.69	747.35	2
Golgin subfamily A member 6A	GNHEGHG	706.28	354.15	2
Collagen alpha-2(IV) chain	EVLGAQPGTRGDAGLPGQPG	1875.93	626.32	3
MTOR-associated protein MEAK7	DVDGLFDTLSGSSSSAAAKNGK	2126.05	1064.03	2
Transforming protein Maf	GSAAAVVSAVIAAA	1156.53	579.27	2
Glycine dehydrogenase (decarboxylating)	PGAMGADIAIG	971.4	486.71	2
L-lactate dehydrogenase A-like 6B	SVADLTESILK	1174.65	392.56	3
CTP synthase	PDGKLVEICEVTGHPF	1739.83	870.92	2
Collagen alpha-3(V) chain	GIPGPLGPL	819.45	410.73	2
T-related protein	VSGGGGGGGAGGGAGSGSPQ	1429.68	715.85	2
Glyceraldehyde-3-phosphate dehydrogenase 1	TVDGPSGK	759.37	380.69	2
UDP-3-O-acylglucosamine N-acyltransferase	ADGFGFAPDFGPQGGEW	1753.78	877.9	2
Protein prickle	GGGAGGSSGGPGGADAAAAPAAGQ	1767.76	884.89	2
Histone H2A	AQGGVLPNIQ	995.54	498.78	2
Putative cuticle collagen 155	GPSGPNGNPGAPGAPGQ	1430.71	716.36	2
BTB/POZ domain and ankyrin repeat-				
containing protein NH5.1	GGAGGGGGAP	656.34	329.18	2
PE-PGRS family protein PE_PGRS5	GAGGKGGNGGTGGAGGPGG	1341.64	671.83	2
Collagen alpha-5(IV) chain	PGIPGIGLPGPPGPKGFPGIP	1947	974.51	2
Glutamate dehydrogenase 1, mitochondrial	IGPGIDVPAPDMSTGE	1554.73	778.37	2
Collagen alpha-2(IV) chain	SGPSGIPGLPGPKGEPGY	1665.76	833.89	2
Collagen alpha-1(I) chain	GLPGSPGPAGEAGK	1193.6	597.81	2
TRPM8 channel-associated factor homolog	SEAVQTNLVPFFEAWGWPI	2190.1	1096.06	2
Collagen alpha-4(IV) chain	GPPGIPGPNGEDGLPGLP	1639.76	820.89	2
Elastin	VPGAVPGGVP	848.44	425.23	2
Multidrug resistance protein PE_PGR46	IMVVVQPFVLVAI	1426.82	714.42	2
Uncharacterized PE-PGRS family protein PE_PGRS46	GDGAPGGDGGAGPLLIGNG	1550.68	776.35	2
POTE ankyrin domain family member E	SGDGVTH	671.29	336.65	2
Actin-related protein 3	SEVVDEVIQN	1130.54	566.28	2
Actin-related protein 3	SGDGVTH	671.29	336.65	2
Protein Wiz	GPERLPGPAPRENIEGGAE	1944.94	973.48	2
DNA-directed RNA polymerase subunit beta	GKPIPESGLPE	1122.53	562.27	2
Histone H2A	AQGGVLPNIQ	995.54	498.78	2
Ribulose bisphosphate carboxylase/oxygenase activase 2, chloroplastic	TLMNIADNPTNVQLP	1639.72	820.87	2
FT-interacting protein 1	PEVFVKAQVGNQILK	1668.86	835.43	2
Collagen alpha-2(I) chain	GAVGPVGPVG	808.44	405.23	2
Collagen alpha-2(I) chain	GPIGPPGNPGA	932.47	467.24	2
Polyribonucleotide nucleotidyltransferase	TEAVVAEGLEAAKP	1383.75	692.88	2
Putative cuticle collagen 145	EGPAGPAGPAGPDGQPGA	1501.64	751.83	2
Contactin-3	VSGGGGSRSELVITWDPVP	1911.97	956.99	2
Collagen alpha-1(III) chain	EPGQAGPAGPPGPPG	1285.6	1286.61	1
Collagen alpha-2(I) chain	SIGEPGPIGIAG	1066.51	534.26	2
Collagen alpha-2(I) chain isoform X3	GDPGPGGPQGEPGAVGPAGITGDKGPSGES	2601.2	868.08	3
Uncharacterized protein	DIKPVTEIQQNGNDFVITSK	2245.16	749.4	3
Calmodulin	IDQLTEEQIAEF	1434.65	718.33	2
Mitochondrial fission regulator	HLSLPRFFPSRTGE	1643.18	548.73	3
Collagen, type V, alpha 3a	LIDVLRVLELSEDMEGVSV	2114.92	1058.47	2
Si:dkey-237h12.3	ELDASNMGGWSLDK	1521.81	761.91	2
Uncharacterized protein *Salmo truta*	AGAEGFDDIK	1021.47	511.74	2
Fatty acid-binding protein, liver	AIGLPDDLIQK	1181.67	591.84	2
Uncharacterized protein *Sinocyclocheilus*				
*anshuiensis*	DVFRDGFTMDT	1302.61	652.31	2
Collagen alpha-4(IV) chain	GSSPIGPPGSPGSPGASGQ	1592.74	797.38	2
Mucin-5AC-like	GGPTSGSEGGDNESIK	1490.65	746.33	2
D-dopachrome decarboxylase	MIVVVKPGLPMLM	1426.82	714.42	2
Uncharacterized protein OS = *Echeneis* *naucrates*	PKPLPFFGTMLSYR	1653	827.51	2
Fumarylacetoacetase	IGVAIGDQILDLSVIK	1652.97	827.49	2

**Table 2:** Peptides identified in salmon viscera extract obtained by conventional stirring.

Protein of Origin of the Identified Peptide	Sequence	Obs MW	Obs *m/z*	Theor z
Adenosylhomocysteinase	GVSEETTTGVH	1115.51	558.76	2
Hemoglobin subunit alpha	AIHFPADFTPEVH	1479.71	494.24	3
Forkhead box protein K1	PQPPPGPPPPP	1076.57	539.29	2
40S ribosomal protein	ADGYEPPIQET	1218.54	610.28	2
WW domain-binding protein 11	PGPPPGPPPP	908.48	455.24	2
Filamin-A	VITPEEIVDPNVDEH	1704.81	569.28	3
Collagen alpha-1(X) chain	ISVPGKPGPQ	978.47	490.24	2
Fatty acid-binding protein 10-A, liver basic	AQENYEEFLR	1297.59	649.8	2
Methionine import ATP-binding protein MetN	IDEIGGQHVGSLVLGVP	1688.81	845.41	2
Probable tRNA pseudouridine synthase	ENNVDFVNRKIKEGEAMVSGPI	2445.24	816.09	3
Mediator of RNA polymerase II transcription subunit 30	LAASGMAPGPFAGPQ	1370.71	686.36	2
1-(5-phosphoribosyl)-5-[(5-phosphoribosylamino)- methylideneamino] imidazole-4-				
carboxamide isomerase	HWVDQGGKRLHL	1444.89	723.45	2
Quinolinate synthase A	EGADEVHVDPGI	1236.58	619.29	2
40S ribosomal protein S17	DQEIIEVDPDT	1272.58	637.3	2
Uncharacterized PE-PGRS family protein	NGGNGGDGGNGGDGGNGAP	1627.66	814.84	2
PE_PGRS54	GPPPPGPPPEVVI	1251.65	626.83	2
Prostaglandin reductase 1	LVGAGNNGGDALLAAAELAR	1851.87	926.94	2
NAD(P)H-hydrate epimerase	VLRFFMATTQYR	1531.9	766.96	2
Cysteine--tRNA ligase	DSGDGVTH	786.32	787.33	1
Actin, cytoplasmic 1	LDRMKNSCIVCNIGH	1701.92	851.97	2
Putative adenosylhomocysteinase 3	SSSSILVVIATL	1188.79	595.4	2
Spore membrane assembly protein 2	IPAINVNDSVT	1141.6	571.81	2
Adenosylhomocysteinase	IHFPADFTPEVH	1408.68	470.57	3
Hemoglobin subunit alpha	VFASYPQPLG	1077.53	539.77	2
Uncharacterized protein y4iR				
2-C-methyl-D-erythritol 4-phosphate-				
cytidylyltransferase	LQSVIAVVPAAGV	1222.84	612.43	2
Zinc finger C2HC domain-containing				
protein 1A	NQVIKDGGPLPPPPPP	1621.8	811.91	2
Trichodiene synthase	VSEGITLNQALE	1272.58	637.3	2
60 kDa heat shock protein, mitochondrial	GTSDVEVNEK	1076.5	539.25	2
pH-response regulator protein palF/RIM8	PIRITHLTVAL	1232.8	617.41	2
Leucine-rich repeat-containing protein 56	LEQLEVLDLEGNS	1457.65	729.83	2
Tungsten-containing formylmethanofuran-				
dehydrogenase 2 subunit C	DVDVRVGGEMKAG	1331.66	666.84	2
Cyclic pyranopterin monophosphate synthase	NTNGEANMVDVSMKQ	1636.8	819.41	2
Acetylcholinesterase	FRHPRPAEKWTGV	1579.88	790.95	2
Uncharacterized PE-PGRS family protein PE	NGGNGGIGGP	798.43	400.22	2
Sulfocyanin	SPSASSSTGTSTGP	1222.59	612.3	2
Actin, cytoplasmic	VMDSGDGVTH	1016.42	509.22	2
40S ribosomal protein S3a	GEGGGSSAAKPSG	1060.47	531.24	2
DNA repair protein crb2	DSLYDRLLARKGPLFGK	1948.23	975.12	2
Argininosuccinate synthase	IEGGRLEDPSFVPP	1511.82	756.92	2
Collagen alpha-2(I) chain	GAVGPVGPVG	808.44	405.23	2
Thiazole synthase	GVLLNTAVSGAKDP	1340.73	671.37	2
Histone-lysine N-methyltransferase 2D	PLSPPPEDSPLSPPP	1525.79	509.61	3
Probable transcriptional regulatory				
protein Ecaj_0351	NFDSLFNIAI	1152.59	577.3	2
50S ribosomal protein L29	HAKKAELFELRVK	1567.85	784.93	2
Forkhead box protein K1	QPPPGPPPPPP	1076.57	539.29	2
Probable GPI-anchored adhesin-like				
protein PGA32	ATAAGTEVQGFTPI	1361.6	681.81	2
Replicase polyprotein 1ab	MAKMGKYGLGFK	1329.9	665.96	2
Stonin-2	VVDGGSQDHS	999.35	500.68	2
SLAIN motif-containing protein	AGGGGPEPGGAGTPPGAAAAP	1615.84	808.93	2
Structural maintenance of chromosomes				
protein 4	EIQNSILNVGGPQ	1367.67	684.84	2
Golgin-84	TPEIH	595.3	298.66	2
Keratin, type II cytoskeletal 5	LGGGAGFGGGYGGP	1122.54	562.28	2
Translation initiation factor IF-2	VEEGLTSDEPDLE	1431.6	716.81	2
Genome polyprotein	IDLSANAAGSDPP	1226.61	614.31	2
Collagen alpha-1(X) chain	ISVPGKPGPQ	978.47	490.24	2
Transcription-associated protein 1	VASVQPYAMPP	1158.53	580.27	2
MAM and LDL-receptor class A domain-				
containing- protein 2	LDDSPCPPE	971.37	972.38	1
Coiled-coil domain-containing protein CG32809	SSSKKKRKGRE	1289.85	645.93	2
Large tegument protein deneddylase	SVPAPPTLPP	974.52	488.27	2
Adenylyl cyclase-associated protein	GPPPPGPPPPP	1004.53	503.27	2
Neuroblast differentiation-associated protein AHNAK	VDIEGPDVDIEGSGG	1457.65	729.83	2
Mediator of RNA polymerase II transcription subunit 28	QPPGPPPPPPP	1076.56	539.29	2
Protein S100	DLDANSDGSVDFQ	1381.56	691.79	2
LisH domain-containing protein	VISYALDLIEVKHDSARVH	2164.32	1083.16	2
Adenylyl cyclase-associated protein	DGDYTEIPVPEQ	1361.59	681.8	2
Guanylate cyclase domain-containing protein	LISPGDAL	784.35	393.18	2
Insulin receptor substrate 2	VCGGSGPG	632.26	317.14	2

**Table 3:** Comparison of peptides identified in salmon viscera extracts obtained through pressurized liquid extraction with potential antioxidant sequences contained in the BIOPEP-UWM database.

Sequence_Modification	Sequence in BIOPEP-UWM Database	Identity of Sequences with Antioxidant Potential
GPAGPHGPPG	PHG	ID 8026 synthetic peptide
	GPP	ID 8987
GPAGHPGPPG	GPP	ID 8987
GYAKDGLPGIPGPQGET	KD	ID 8134 peptide from dried bonito
GGGEGYGGGGANGGGY	GGE	ID 8114 peptide from sardinella byproducts
GPLGPPGGMPGH	GPP	ID 8987
GPPGLPGPPGPPGHKGF_ Carbamyl(K)@15	GPP	ID 8987
GGGGGGGGGGGGSGGNFGGGGPP	GPP	ID 8987
QPPPGPPPPPP	GPP	ID 8987
TALGGAAGGMGGGGGMGGGM_ Oxidation(M)@20	GAA	ID 8983
ACAGMIGPPGPQGFP_ Deamidated(Q)@12	GPP	ID 8987
	ACA	ID 10038
QAGEGGAGAGAGAAG	GAA	ID 8983
LPGPPGPPGPPGPRGYPG	GPP	ID 8987
AIQPDTEFTPPELDASS	EL	ID 7888
	PEL	ID 8139 synthetic peptide
	GPP	ID 8987
LSLVVSGGHTELVL	EL	ID 7888
QNLVGPPGPPGPPGVSGD_ Gln->pyro-Glu@N-term	GPP	ID 8987
DINAGGGACASVGLL	ACA	ID 10038
KGDRFLEAAGVNKLWPE	LW	ID 8462 peptide from marine bivalve
RGDGGPPGVTGFPGAA	GAA	ID 8983
	GPP	ID 8987
GPAGPHGPP	PHG	ID 8026
	GPP	ID 8987
ETNPADSKPGSI	KP	ID 8218
NGGAAGLIGNGGAGGAGGAGGAG	GAA	ID 8983
DQGLFMGPSTSGAAANR_ Deamidated(N)@16	GAA	ID 8983
GIADKQMSELSCHA	EL	ID 7888
DALGLELNADE	EL	ID 7888
DGNPGLPGPPGPPGPPG_ Pro->pyro-Glu(P)@16	GPP	ID 8987
SVADLTESILK	LK	ID 8217
ADGFGFAPDFGPQGGEW	GGE	ID 8114 peptide from sardinella by-products
	ADGF	ID 9328
PGIPGIGLPGPPGPKGFPGIP_ Delta:H(2)C(2)(K)@15	GPP	ID 8987
SEAVQTNLVPFFEAWGWPI	WG	ID 9082
	EAVQ	ID 9881
GPPGIPGPNGEDGLPGLP	GPP	ID 8987
GKPIPESGLPE	KP	ID 8218
PEVFVKAQVGNQILK	LK	ID 8217
GPIGPPGNPGA	GPP	ID 8987
TEAVVAEGLEAAKP	KP	ID 8218
VSGGGGSRSELVITWDPVP	EL	ID 7888
	TW	ID 8459 peptide from marine bivalve
EPGQAGPAGPPGPPG_ Deamidated(Q)@4	GPP	ID 8987
DIKPVTEIQQNGNDFVITSK	KP	ID 8218
HLSLPRFFPSRTGE	HL	ID 3317
LIDVLRVLELSEDMEGVSV	EL	ID 7888
ELDASNMGGWSLDK	EL	ID 7888
GGPTSGSEGGDNESIK	GPP	ID 8987
MIVVVKPGLPMLM	KP	ID 8218
	VKP	ID 8434 peptide from jellyfish
PKPLPFFGTMLSYR	LPM	ID 9360
IGVAIGDQILDLSVIK	KP	ID 8218

**Table 4:** Comparison of peptides identified in salmon viscera extract obtained through conventional stirring with potential antioxidant sequences contained in the BIOPEP-UWM database.

Sequence_Modification	Sequence in BIOPEP-UWM Database	Identity of Sequences with Antioxidant Potential
AIHFPADFTPEVH	ADF	ID 7868 peptide from Okara protein
PQPPPGPPPPP	GPP	ID 8987
PGPPPGPPPP	GPP	ID 8987
ISVPGKPGPQ	KP	ID 8218
HWVDQGGKRLHL	LH	ID 3305
	HL	ID 3317
	LHL	ID 7995 synthetic peptide
GPPPPGPPPEVVI	GPP	ID 8987
LVGAGNNGGDALLAAAELAR	EL	ID 7888
IHFPADFTPEVH	ADF	ID 7868 peptide from Okara protein
NQVIKDGGPLPPPPPP	KD	ID 8134 peptide from dried bonito
PIRITHLTVAL	HL	ID 3317
	IR	ID 8215
DVDVRVGGEMKAG	GGE	ID 8114 peptide from sardinella by-products
GEGGGSSAAKPSG	KP	ID 8217
GVLLNTAVSGAKDP	KD	ID 8134 peptide from dried bonito
HAKKAELFELRVK	EL	ID 7888
QPPPGPPPPPP	GPP	ID 8987
AGGGGPEPGGAGTPPGAAAAP	GAA	ID 8983

**Table 5:** Concentration of heavy metals in salmon side streams.

Salmon Side Streams	Heavy Metals (µg/g of Wet Weight)			
	As	Hg	Cd	Pb
Muscle	0.5413 ± 0.0068	0.0238 ± 0.0005	0.0004 ± 0.0001	0.0269 ± 0.0002
Head	0.6922 ± 0.0072	0.0157 ± 0.0005	0.0011 ± 0.0001	0.0190 ± 0.0001
Viscera	0.4617 ± 0.0055	0.0095 ± 0.0002	0.0044 ± 0.0002	0.0071 ± 0.0001
Skin	0.4504 ± 0.0032	0.0077 ± 0.0003	0.0019 ± 0.0001	0.0247 ± 0.0001
Tailfin	0.4186 ± 0.0054	0.0408 ± 0.0015	0.0104 ± 0.0003	0.0859 ± 0.0016
(Legislation *)	<13.5	<0.50	<0.05	<0.30

* values refer to fish muscle tissue [23,25,35].

## Data Availability

Not applicable.
